# Safety profile of vunakizumab in elderly patients with moderate-to-severe plaque psoriasis: a *post-hoc* analysis

**DOI:** 10.3389/fmed.2025.1695266

**Published:** 2026-01-12

**Authors:** Tong Mu, Yuanxiong He, Min Zhang, Chunyou Wang, Zhifang Zhai, Zhiqiang Song

**Affiliations:** Department of Dermatology, The First Affiliated Hospital (Southwest Hospital) of Army Medical University, Chongqing, China

**Keywords:** efficacy, elderly patients, moderate-to-severe plaque psoriasis, safety, vunakizumab

## Abstract

**Objective:**

Elderly patients with moderate-to-severe plaque psoriasis are vulnerable to adverse events (AEs), which raise safety concerns in their management. This *post-hoc* analysis aimed to evaluate the safety and efficacy of vunakizumab in elderly patients with moderate-to-severe plaque psoriasis, with an emphasis on safety.

**Methods:**

Data were extracted from a phase III trial (NCT04839016). A total of 70 elderly patients with moderate-to-severe plaque psoriasis were included and assigned to the vunakizumab (*n* = 49) and placebo (*n* = 21) groups.

**Results:**

During the induction period, the incidence of any AEs (63.3% vs. 76.2%) (*p* = 0.291) and serious AEs (4.1% vs. 0.0%) (*p* = 1.000) did not differ between the vunakizumab and placebo groups. During the entire treatment period, the incidence of any AEs (87.8% vs. 90.5%) (*p* = 1.000) and serious AEs (10.2% vs. 4.8%) (*p* = 0.661) remained unchanged between the vunakizumab and placebo groups. In the vunakizumab group, the most frequent AEs during the entire treatment period were elevated blood glucose (22.4%), hypertriglyceridemia (12.2%), elevated blood cholesterol (10.2%), eczema (10.2%), hyperuricemia (10.2%), and upper respiratory tract infection (10.2%). The rates of patients achieving psoriasis area and severity index (PASI) 75, 90, 100, and static physician’s global assessment (sPGA) 0/1 responses at week (W) 12 were higher in the vunakizumab group than in the placebo group (all *p* < 0.001). These trends were sustained for 52 weeks. Patient-reported outcomes (PROs) at certain time points during 52 weeks were better in the vunakizumab group than in the placebo group.

**Conclusion:**

Vunakizumab is safe and effective in elderly patients with moderate-to-severe plaque psoriasis.

## Introduction

Psoriasis is a complex autoimmune skin disease, with the greatest disease burden among the elderly (1). Approximately 15% of elderly patients have moderate-to-severe plaque psoriasis, and systemic treatments are recommended for these patients (2–6). Nevertheless, elderly patients with moderate-to-severe plaque psoriasis are susceptible to adverse events (AEs) during systemic treatments, which can be attributed to age-related physiological changes leading to altered drug metabolism and a high prevalence of comorbidities, thereby raising safety concerns (5, 7). Additionally, the cost of biologic therapies is a significant consideration for elderly patients, who may face financial constraints or limited insurance coverage, potentially impacting treatment access and adherence (4). Given the unique safety considerations in elderly patients with moderate-to-severe plaque psoriasis, investigating safe and effective systemic treatments is particularly important for this subset of patients.

Vunakizumab, a novel humanized IgG1/*κ* monoclonal antibody that selectively targets interleukin (IL)-17A, has received approval for the treatment of moderate-to-severe plaque psoriasis in China (8). In the prior phase III trial (NCT04839016), vunakizumab demonstrated favorable efficacy for treating patients with moderate-to-severe plaque psoriasis (9). Notably, the safety profile of vunakizumab was good in these patients; during the induction period, the occurrence rate of AEs (69.1% vs. 71.6%) was comparable between the vunakizumab and placebo groups; possible treatment-related serious adverse events (SAEs) occurred in 0.9% of vunakizumab-treated subjects. In addition, throughout the whole treatment period, vunakizumab showed satisfactory tolerability (9). However, its safety and efficacy specifically in elderly patients (≥60 years) remain unexplored. Thus, this *post-hoc* analysis aimed to address this gap by evaluating vunakizumab in elderly patients with an emphasis on safety.

## Methods

### Study design and population

Data were extracted from a phase III trial (NCT04839016), which enrolled 690 patients. This study comprised a 12-week, double-blinded, placebo-controlled induction treatment period, followed by a 40-week, double-blinded maintenance period, and a 16-week safety follow-up period. Enrolled subjects were randomized (2:1) to receive vunakizumab 240 mg or a matching placebo subcutaneously at weeks 0, 2, 4, and 8. At week 12, subjects initially assigned placebo were switched to receive vunakizumab 240 mg (weeks 12, 14, 16, and every 4 weeks thereafter), and other subjects continued vunakizumab every 4 weeks through week 52 (9).

For this *post-hoc* analysis, elderly patients (aged ≥60 years) were identified as a subgroup of the original phase III trial. There were no additional exclusion criteria apart from the phase III trial. Therefore, a total of 70 elderly patients were selected, among whom 49 were in the vunakizumab group, and 21 were in the placebo group.

### Ethics and data

The original phase III trial was performed in accordance with the Declaration of Helsinki, the International Conference on Harmonization Good Clinical Practice guideline, and applicable regulatory requirements. The protocol and all amendments were approved by the ethics committee at each site. All subjects provided written informed consent (9). This *post-hoc* analysis utilized anonymized data for secondary data analysis.

Baseline demographics, prior histories, and disease-related information were screened and summarized descriptively. Additionally, safety results, efficacy indicators, and patient-reported outcomes (PROs) were also extracted for analysis.

### Safety and efficacy assessment

Adverse events (AEs) during the induction period (first 12 weeks) and the entire 52-week treatment period were assessed for safety. Any AEs and the AEs that exceeded 5% in the vunakizumab group reported in the original phase III study were listed. Efficacy outcomes included the proportions of patients achieving Psoriasis Area and Severity Index (PASI) 75, 90, and 100 responses, and a static Physician’s Global Assessment (sPGA) score of 0 or 1. PASI 75, 90, and 100 were defined as at least a 75, 90%, and complete improvement in PASI score compared to baseline, respectively. sPGA 0/1 indicated that the sPGA score achieved 0 or 1. The timepoints for efficacy assessment included 2 (W2), 4 (W4), 8 (W8), 12 (W12), 14 (W14), 16 (W16), 20 (W20), 24 (W24), 28 (W28), 32 (W32), 36 (W36), 40 (W40), 44 (W44), 48 (W48), and 52 (W52) weeks after treatment initiation. The PASI 75, 90, 100, and sPGA 0/1 at W12 were the co-primary endpoints of this *post-hoc* analysis.

### Quality of life

The PROs information was screened to evaluate the quality of life. PROs included the Dermatology Life Quality Index (DLQI) (10), Itch Numeric Rating Scale (I-NRS) (28), EuroQol-5D (EQ-5D) utility index, EQ-5D Visual Analog Scale (VAS), and Short Form-36 (SF-36) scores (11). The timepoints for PRO evaluation included W4, W8, W12, W20, W28, W36, W44, and W52.

### Statistical analysis

Statistical analysis for this *post-hoc* analysis was performed from April to May 2025. SPSS software (version 29.0, IBM, United States) was used for data processing. The *t*-test, *χ*^2^, or Fisher’s exact test was utilized for comparison analysis. Time-to-PASI 75, 90, or 100 response was evaluated via Kaplan–Meier curves, in which the data between groups were analyzed using the log-rank test. The principle for handling missing data was consistent with the original phase III trial (9). All tests were two-sided with *α* = 0.05.

## Results

### Clinical characteristics

The mean age was 66.0 ± 4.5 years in the vunakizumab group and 65.3 ± 5.9 years in the placebo group. There were 10 (20.4%) women and 39 (79.6%) men in the vunakizumab group, as well as 3 (14.3%) women and 18 (85.7%) men in the placebo group. A total of 12 (24.5%) patients in the vunakizumab group and 8 (38.1%) patients in the placebo group had a history of hyperlipidemia. No patients had a history of cardiovascular diseases. All baseline characteristics did not differ between the two groups (all *p* > 0.05) (Table 1).

**Table 1:** Characteristics of elderly moderate-to-severe chronic plaque psoriasis patients.

Items	Vunakizumab (*N* = 49)	Placebo (*N* = 21)	*p*-values
Age (years), mean ± SD	66.0 ± 4.5	65.3 ± 5.9	0.604
Sex, *n* (%)			0.741
Female	10 (20.4)	3 (14.3)	
Male	39 (79.6)	18 (85.7)	
BMI, *n* (%)			0.075
<24 kg/m^2^	19 (38.8)	13 (61.9)	
≥24 kg/m^2^	30 (61.2)	8 (38.1)	
Smoking, *n* (%)			0.834
Never	27 (55.1)	11 (52.4)	
Former or current	22 (44.9)	10 (47.6)	
Family history of psoriasis, *n* (%)			0.434
No	45 (91.8)	18 (85.7)	
Yes	4 (8.2)	3 (14.3)	
Previous therapy, *n* (%)			1.000
No	2 (4.1)	0 (0.0)	
Yes	47 (95.9)	21 (100.0)	
Hypertension, *n* (%)			0.201
No	27 (55.1)	15 (71.4)	
Yes	22 (44.9)	6 (28.6)	
Hyperlipidemia, *n* (%)			0.248
No	37 (75.5)	13 (61.9)	
Yes	12 (24.5)	8 (38.1)	
DM, *n* (%)			0.094
No	42 (85.7)	21 (100.0)	
Yes	7 (14.3)	0 (0.0)	
Duration of psoriasis (years), mean ± SD	18.6 ± 13.1	16.6 ± 13.6	0.563
PASI score, *n* (%)			0.064
≤30	41 (83.7)	13 (61.9)	
>30	8 (16.3)	8 (38.1)	
sPGA score, *n* (%)			0.191
<4	17 (34.7)	4 (19.0)	
≥4	32 (65.3)	17 (81.0)	
BSA involved (%), mean ± SD	34.7 ± 16.6	41.3 ± 19.1	0.155
DLQI score, mean ± SD	9.7 ± 7.8	10.9 ± 8.7	0.573
I-NRS score, mean ± SD	6.2 ± 2.4	6.8 ± 3.3	0.456
EQ-5D utility index, mean ± SD	0.9 ± 0.1	0.9 ± 0.1	0.997
EQ-5D VAS score, mean ± SD	83.7 ± 13.2	79.3 ± 16.2	0.237
SF-36 MCS, mean ± SD	51.3 ± 11.1	49.6 ± 11.2	0.566
SF-36 PCS, mean ± SD	51.5 ± 6.4	51.5 ± 7.9	0.982

SD, standard deviation; BMI, body mass index; DM, diabetes mellitus; PASI, psoriasis area and severity index; sPGA, static physician’s global assessment; BSA, body surface area; DLQI, Dermatology Life Quality Index; I-NRS, Itch Numerical Rating Scale; EQ-5D, EuroQol-5D; VAS, Visual Analogic Scale; SF-36, Short Form-36; MCS, mental component score; PCS, physical component score.

### Safety

During the induction period, the incidence of any AEs did not differ between the vunakizumab and placebo groups (63.3% vs. 76.2%) (*p* = 0.291). Additionally, the incidence of SAEs was comparable between the vunakizumab and placebo groups (4.1% vs. 0.0%) (*p* = 1.000). The incidence of specific AEs was also not different between the two groups (all *p* > 0.05). The most common AEs during the induction period in the vunakizumab group were elevated blood glucose (12.2%), elevated blood cholesterol (6.1%), hypercholesterolemia (6.1%), hypertriglyceridemia (6.1%), and hyperuricemia (6.1%). The incidence of these AEs in the placebo group was 14.3, 0.0, 0.0, 0.0, and 14.3%, respectively.

During the entire treatment period, the incidence of any AEs was not different between the vunakizumab and placebo groups (87.8% vs. 90.5%) (*p* = 1.000). No significant difference in the incidence of SAEs was observed between the vunakizumab group and placebo group (10.2% vs. 4.8%) (*p* = 0.661). There was no difference in the incidence of specific AEs between the two groups (all *p* > 0.05). The most common AEs during the entire treatment period were elevated blood glucose (22.4%), hypertriglyceridemia (12.2%), elevated blood cholesterol (10.2%), eczema (10.2%), hyperuricemia (10.2%), and upper respiratory tract infection (URTI) (10.2%). The incidence of these AEs in the placebo group was 19.0, 0.0, 0.0, 9.5, 14.3, and 19.0%, respectively (Table 2).

**Table 2:** AEs.

AEs, *n* (%)	Induction period			Entire treatment period		
	Vunakizumab (*N* = 49)	Placebo (*N* = 21)	*p*-value	Vunakizumab (*N* = 49)	Placebo (*N* = 21)	*p*-value
Any	31 (63.3)	16 (76.2)	0.291	43 (87.8)	19 (90.5)	1.000
SAEs	2 (4.1)	0 (0.0)	1.000	5 (10.2)	1 (4.8)	0.661
Elevated ALT	2 (4.1)	1 (4.8)	1.000	4 (8.2)	1 (4.8)	1.000
Elevated AST	0 (0.0)	1 (4.8)	0.300	2 (4.1)	1 (4.8)	1.000
Elevated blood bilirubin	0 (0.0)	2 (9.5)	0.087	3 (6.1)	3 (14.3)	0.355
Elevated blood cholesterol	3 (6.1)	0 (0.0)	0.549	5 (10.2)	0 (0.0)	0.313
Elevated blood glucose	6 (12.2)	3 (14.3)	1.000	11 (22.4)	4 (19.0)	1.000
Elevated blood TG	2 (4.1)	0 (0.0)	1.000	3 (6.1)	0 (0.0)	0.549
Elevated blood UA	2 (4.1)	0 (0.0)	1.000	3 (6.1)	1 (4.8)	1.000
Elevated LDL	2 (4.1)	0 (0.0)	1.000	4 (8.2)	0 (0.0)	0.309
Eczema	2 (4.1)	0 (0.0)	1.000	5 (10.2)	2 (9.5)	1.000
Hypercholesterolemia	3 (6.1)	0 (0.0)	0.549	4 (8.2)	0 (0.0)	0.309
Hyperlipidemia	1 (2.0)	2 (9.5)	0.212	3 (6.1)	3 (14.3)	0.355
Hypertriglyceridemia	3 (6.1)	0 (0.0)	0.549	6 (12.2)	0 (0.0)	0.168
Hyperuricemia	3 (6.1)	3 (14.3)	0.355	5 (10.2)	3 (14.3)	0.689
Injection site reaction	1 (2.0)	0 (0.0)	1.000	4 (8.2)	0 (0.0)	0.309
Pruritus	2 (4.1)	2 (9.5)	0.578	4 (8.2)	2 (9.5)	1.000
URTI	0 (0.0)	0 (0.0)	(−)	5 (10.2)	4 (19.0)	0.437
Urticaria	1 (2.0)	0 (0.0)	1.000	2 (4.1)	2 (9.5)	0.578

AEs, adverse events; SAEs, serious adverse events; ALT, alanine aminotransferase; AST, aspartate aminotransferase; TG, triglycerides; UA, uric acid; LDL, low-density lipoprotein; URTI, upper respiratory tract infection.

### Treatment response

**Figure 1:**
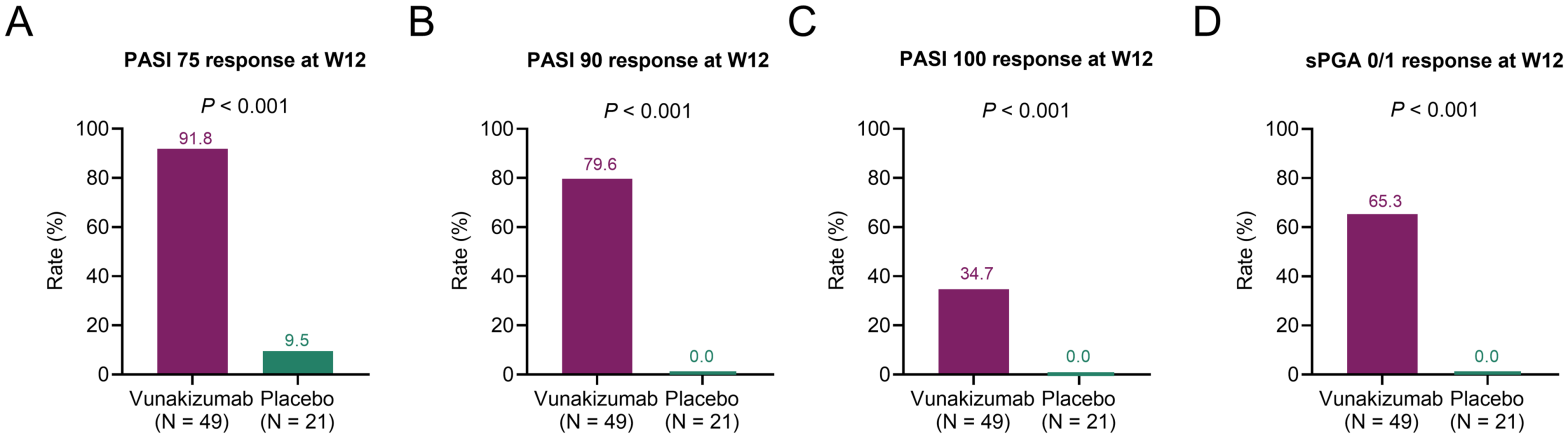
Comparison of treatment response at W12. Comparison of the rates of patients achieving PASI 75 **(A)**, PASI 90 **(B)**, PASI 100 **(C)**, and sPGA 0/1 **(D)** responses at W12 between the vunakizumab and placebo groups. The *χ*^2^ and Fisher’s exact test were used.

**Figure 2:**
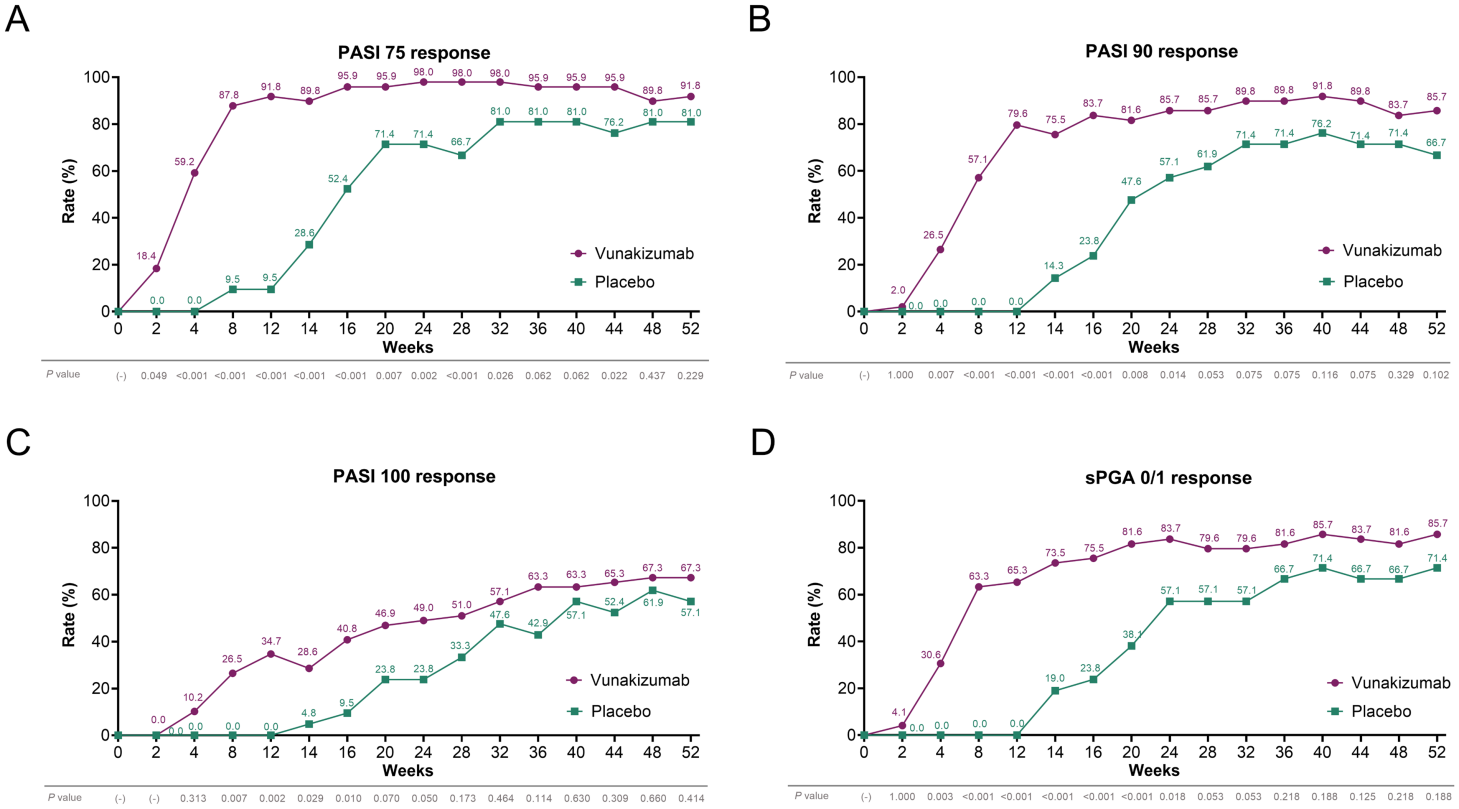
Comparison of treatment response during 52 weeks. Comparison of the rates of patients achieving PASI 75 **(A)**, PASI 90 **(B)**, PASI 100 **(C)**, and sPGA 0/1 **(D)** responses during 52 weeks between the vunakizumab and placebo groups. The *χ*^2^ and Fisher’s exact test were used.

**Figure 3:**
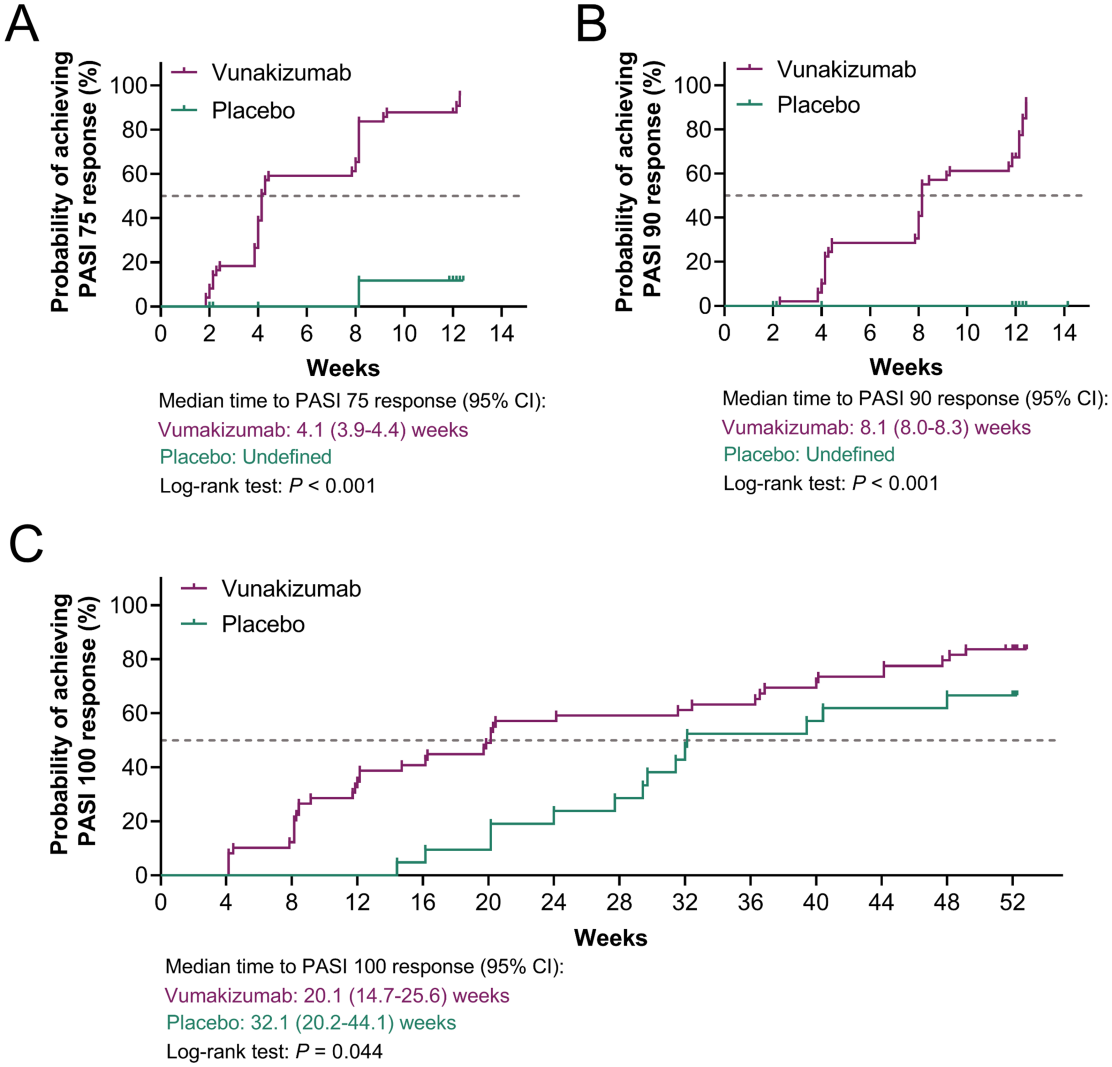
Comparison of the overall achievement of treatment response. Comparison of the overall achievement of PASI 75 **(A)**, PASI 90 **(B)**, and PASI 100 **(C)** responses between the vunakizumab and placebo groups. The log-rank test was used.

The rates of patients achieving PASI 75 (91.8% vs. 9.5%) (Figure 1A), PASI 90 (79.6% vs. 0.0%) (Figure 1B), PASI 100 (34.7% vs. 0.0%) (Figure 1C), and sPGA 0/1 (65.3% vs. 0.0%) (Figure 1D) responses at W12 were significantly higher in the vunakizumab group than in the placebo group (all *p* < 0.001). During 52 weeks, the rates of patients achieving the PASI 75 response at most time points were significantly higher in the vunakizumab group than in the placebo group (*p* < 0.05) (Figure 2A). The rates of patients achieving PASI 90 (Figure 2B), PASI 100 (Figure 2C), and sPGA 0/1 (Figure 2D) responses at various time points over 52 weeks were significantly higher in the vunakizumab group than in the placebo group (*p* < 0.05). The vunakizumab group showed significantly better PASI75, PASI 90, and PASI 100 response probabilities than the placebo group at the corresponding timeframes (all *p* < 0.05). The median [95% confidence interval (CI)] time to achieve PASI 75, PASI 90, and PASI 100 was 4.1 (3.9–4.4), 8.1 (8.0–8.3) weeks, and 20.1 (14.7–25.6) weeks in the vunakizumab group (Figures 3A–C).

### PROs

**Figure 4:**
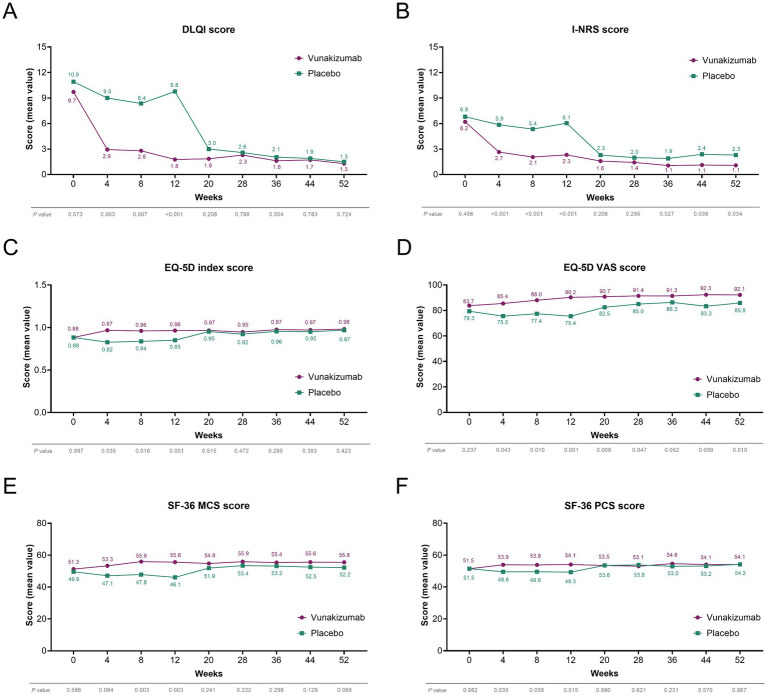
Comparison of PROs during 52 weeks. Comparison of DLQI score **(A)**, I-NRS score **(B)**, EQ-5D index score **(C)**, EQ-5D VAS score **(D)**, SF-36 MCS **(E)**, and SF-36 PCS **(F)** during 52 weeks between the vunakizumab and placebo groups. A *t*-test was used.

DLQI scores at W4, W8, and W12 were significantly lower in the vunakizumab group than in the placebo group (all *p* < 0.01) (Figure 4A). I-NRS scores at W4, W8, W12, W36, W44, and W52 were significantly lower in the vunakizumab group than in the placebo group (all *p* < 0.05) (Figure 4B). EQ-5D index scores at W4, W8, and W12 (Figure 4C), as well as EQ-5D VAS scores at W4, W8, W12, W20, W28, W44, and W52 (Figure 4D), were significantly higher in the vunakizumab group than in the placebo group (all *p* < 0.05). SF-36 mental component scores (MCSs) at W8 and W12 (Figure 4E), and SF-36 physical component scores (PCSs) at W4 and W12 (Figure 4F) were significantly higher in the vunakizumab group than in the placebo group. After W12, no significant differences were observed in DLQI scores, EQ-5D index scores, SF-36 MCSs, and SF-36 PCSs between the vunakizumab group and the placebo group.

## Discussion

The previous phase II and III trials have demonstrated the favorable efficacy and safety of vunakizumab in patients with moderate-to-severe plaque psoriasis (9, 12). This *post-hoc* analysis further investigated vunakizumab as a potential treatment for elderly patients, with a particular focus on safety in this subset. Several findings were observed: (1) vunakizumab did not raise any safety concerns during the induction period or the entire treatment period. (2) Vunakizumab achieved satisfactory treatment responses. (3) Vunakizumab improved quality of life, reduced pruritus, enhanced mental health, and strengthened physical function at certain time points over 52 weeks.

Elderly patients with moderate-to-severe plaque psoriasis are generally vulnerable and susceptible to AEs (7). Therefore, safety is a major concern in the management of these patients (3, 13, 14). Some previous studies have shown that IL-17 inhibitors have good safety profiles in elderly patients with moderate-to-severe plaque psoriasis (15–23). In the current *post-hoc* analysis, we found that the incidence of any AEs and SAEs during the induction period and the entire treatment period did not differ between the vunakizumab group and the placebo group. These results suggest that vunakizumab is safe in elderly patients with moderate-to-severe plaque psoriasis. Furthermore, we found that the incidence of any AEs in the vunakizumab group was 63.3% during the induction period and 87.8% throughout the entire treatment period. These findings were comparable to previous studies, which reported that the incidence of any AEs ranged from 47.2 to 82.1% after secukinumab treatment in elderly patients (17, 23). Moreover, the most frequent AEs during the induction period and the entire treatment period after vunakizumab treatment were elevated blood glucose, elevated blood cholesterol, hypertriglyceridemia, and hyperuricemia. Therefore, it is advisable to monitor these laboratory parameters during treatment. These findings were inconsistent with previous studies conducted in other regions (17–19, 21, 22), which may be attributable to the disparities in some factors, such as dietary habits, across regions. Of note, in the prior phase III trial, the most common AEs after vunakizumab treatment included hyperuricemia, URTI, hyperlipidemia, and hypertriglyceridemia (9). Compared with the overall trial population, the types of common AEs in elderly patients differed. Therefore, we speculate that in elderly patients with moderate-to-severe plaque psoriasis, elevated blood glucose and elevated blood cholesterol may require particular attention.

IL-17 inhibitors demonstrate promising efficacy in elderly patients with psoriasis (24). In this *post-hoc* analysis, we found that the rates of patients achieving PASI 75/90/100 and sPGA 0/1 responses at W12 were higher in the vunakizumab group than in the placebo group; additionally, these trends were sustained at several time points during the 52 weeks. Our findings suggest that vunakizumab achieves a favorable treatment response in elderly patients with moderate-to-severe plaque psoriasis. Therefore, vunakizumab could be recommended in this subtype of patients. Notably, the median time to achieve PASI 75 was 4.1 weeks, indicating a rapid onset of action. This swift response is clinically valuable for elderly patients suffering from pruritus and skin lesions, as it can enhance early treatment confidence and adherence. In this *post-hoc* analysis, PASI 75/90/100 and sPGA 0/1 response rates at W12 were 91.8, 79.6, 34.7, and 65.3% in the vunakizumab group. These findings were consistent with prior research focusing on elderly patients with psoriasis (17–19, 21). As reported by a previous study, secukinumab achieved W16 PASI 75/90/100 responses of 86.4, 72.7, and 40.9% (17). Another study reported that PASI 75/90/100 response rates at W12 were 93.7, 87.5, and 62.5% after ixekizumab treatment (18, 19). With respect to the sPGA 0/1 response rate, it was achieved in 70.0% of patients between 3 and 6 months after IL-17 inhibitor treatments (22). Compared with these previous studies, vunakizumab appears to have comparable efficacy to other IL-17 inhibitors in elderly patients with moderate-to-severe plaque psoriasis.

PROs provide important insights into patients’ personal attitudes toward their health status and treatment outcomes, which are essential for the holistic management of psoriasis (25, 26). Assessing PROs in elderly patients with psoriasis is particularly important, as quality of life, mental health, and physical function are unsatisfactory in this population (27). Fortunately, previous studies reported that IL-17 inhibitors improved PROs in elderly patients with moderate-to-severe plaque psoriasis (15, 20). In this *post-hoc* analysis, we observed that DLQI and I-NRS scores at certain time points were lower, while EQ-5D index, EQ-5D VAS, and SF-36 MCS/PCS scores at certain points were higher in the vunakizumab group than in the placebo group. Our findings indicate that, during the induction period (0 to 12 weeks), vunakizumab can improve quality of life, relieve pruritus, enhance mental health, and strengthen physical function to some extent in elderly patients with moderate-to-severe plaque psoriasis.

This *post-hoc* analysis has several limitations. (1) The sample size of this elderly subgroup was limited, which is a common characteristic of *post-hoc* analyses derived from larger trials, and might affect the reliability of our findings. Nevertheless, the data were derived from a rigorously conducted phase III randomized controlled trial, ensuring high data quality. (2) The medical and comorbidity conditions of elderly patients with moderate-to-severe plaque psoriasis are complex in clinical practice. Therefore, real-world clinical studies should be performed to validate the safety and efficacy of vunakizumab in this special population. (3) The safety of vunakizumab in Chinese elderly patients with moderate-to-severe plaque psoriasis was satisfactory. However, to support its wide application, validation on elderly patients from other regions is required. (4) Safety is a major concern in the treatment of elderly patients. Therefore, studies with an extended follow-up duration should be performed to validate the safety of vunakizumab in elderly patients with moderate-to-severe plaque psoriasis.

In conclusion, vunakizumab demonstrates good safety and efficacy in elderly patients with moderate-to-severe plaque psoriasis. In clinical practice, vunakizumab may be considered for use in this patient subgroup. Treatment decisions should be individualized based on comorbidities, patient preferences, and further long-term evidence.

## Data Availability

Publicly available datasets were analyzed in this study. This data can be found here: Yan et al. (9).

## References

[1] Damiani G Bragazzi NL Karimkhani Aksut C Wu D Alicandro G McGonagle D . The global, regional, and national burden of psoriasis: results and insights from the Global Burden of Disease 2019 Study. Front Med. (2021) 8:743180. doi: 10.3389/fmed.2021.743180, 34977058 PMC8716585

[2] Armstrong AW Read C. Pathophysiology, clinical presentation, and treatment of psoriasis: a review. JAMA. (2020) 323:1945–60. doi: 10.1001/jama.2020.4006, 32427307

[3] Di Cesare A Ricceri F Rosi E Fastame MT Prignano F. Therapy of PsO in special subsets of patients. Biomedicine. (2022) 10:2879. doi: 10.3390/biomedicines10112879, 36359399 PMC9687729

[4] Ferrara F Verduci C Laconi E Mangione A Dondi C del Vecchio M . Current therapeutic overview and future perspectives regarding the treatment of psoriasis. Int Immunopharmacol. (2024) 143:113388. doi: 10.1016/j.intimp.2024.113388, 39405929

[5] Megna M Potestio L Fabbrocini G Camela E. Treating psoriasis in the elderly: biologics and small molecules. Expert Opin Biol Ther. (2022) 22:1503–20. doi: 10.1080/14712598.2022.2089020, 35695241

[6] Sbidian E Chaimani A Guelimi R Garcia-Doval I Hua C Hughes C . Systemic pharmacological treatments for chronic plaque psoriasis: a network meta-analysis. Cochrane Database Syst Rev. (2023) 2023:CD011535. doi: 10.1002/14651858.CD011535.pub6, 37436070 PMC10337265

[7] Di Caprio R Caiazzo G Cacciapuoti S Fabbrocini G Scala E Balato A. Safety concerns with current treatments for psoriasis in the elderly. Expert Opin Drug Saf. (2020) 19:523–31. doi: 10.1080/14740338.2020.1728253, 32056449

[8] Keam SJ. Vunakizumab: first approval. Drugs. (2024) 84:1481–5. doi: 10.1007/s40265-024-02110-8, 39497021

[9] Yan K Li F Bi X Han L Zhang Z Chen R . Efficacy and safety of vunakizumab in moderate-to-severe chronic plaque psoriasis: a randomized, double-blind, placebo-controlled phase 3 trial. J Am Acad Dermatol. (2025) 92:92–9. doi: 10.1016/j.jaad.2024.09.031, 39332633

[10] Finlay AY Khan GK. Dermatology Life Quality Index (DLQI)—a simple practical measure for routine clinical use. Clin Exp Dermatol. (1994) 19:210–6. doi: 10.1111/j.1365-2230.1994.tb01167.x, 8033378

[11] Laucis NC Hays RD Bhattacharyya T. Scoring the SF-36 in orthopaedics: a brief guide. J Bone Joint Surg Am. (2015) 97:1628–34. doi: 10.2106/JBJS.O.00030, 26446970 PMC5029523

[12] Zhang C Yan K Diao Q Guo Q Jin H Yang S . A multicenter, randomized, double-blinded, placebo-controlled, dose-ranging study evaluating the efficacy and safety of vunakizumab in patients with moderate-to-severe plaque psoriasis. J Am Acad Dermatol. (2022) 87:95–102. doi: 10.1016/j.jaad.2022.01.005, 35026342

[13] Balato N Patruno C Napolitano M Patrì A Ayala F Scarpa R. Managing moderate-to-severe psoriasis in the elderly. Drugs Aging. (2014) 31:233–8. doi: 10.1007/s40266-014-0156-6, 24554398

[14] Sandhu VK Ighani A Fleming P Lynde CW. Biologic treatment in elderly patients with psoriasis: a systematic review. J Cutan Med Surg. (2020) 24:174–86. doi: 10.1177/1203475419897578, 31950853

[15] Dattola A Bernardini N Caldarola G Coppola R de Simone C Giordano D . Effectiveness of ixekizumab in elderly patients for the treatment of moderate-to-severe psoriasis: results from a multicenter, retrospective real-life study in the Lazio region. Dermatol Pract Concept. (2024) 14:e2024166. doi: 10.5826/dpc.1403a166, 39122514 PMC11314344

[16] Fratton Z Maione V Bighetti S Bettolini L Arisi M Stinco G . Real-world experience of bimekizumab in an elderly patients cohort with plaque-type psoriasis: a 24-week retrospective study. Clin Cosmet Investig Dermatol. (2024) 17:2177–81. doi: 10.2147/CCID.S487869, 39372261 PMC11451462

[17] Körber A Papavassilis C Bhosekar V Reinhardt M. Efficacy and safety of secukinumab in elderly subjects with moderate to severe plaque psoriasis: a pooled analysis of phase III studies. Drugs Aging. (2018) 35:135–44. doi: 10.1007/s40266-018-0520-z, 29404966 PMC5847154

[18] Megna M Camela E Cinelli E Fabbrocini G. Real-life efficacy and safety of secukinumab in elderly patients with psoriasis over a 2-year period. Clin Exp Dermatol. (2020) 45:848–52. doi: 10.1111/ced.14258, 32363583

[19] Megna M Cinelli E Balato A Gallo L Fabbrocini G. Efficacy and safety of ixekizumab in a group of 16 elderly patients with psoriasis over a 1-year period. J Eur Acad Dermatol Venereol. (2020) 34:e152–3. doi: 10.1111/jdv.16063, 31715054

[20] Orsini D Graceffa D Burlando M Campanati A Campione E Guarneri C . Effectiveness, speed of action and safety of brodalumab in elderly psoriasis patients: a multicenter real-world study—IL PSO (Italian Landscape Psoriasis). J Dermatolog Treat. (2025) 36:2452948. doi: 10.1080/09546634.2025.2452948, 39914798

[21] Orsini D Megna M Assorgi C Balato A Balestri R Bernardini N . Efficacy and safety of bimekizumab in elderly patients: real-world multicenter retrospective study—IL PSO (Italian Landscape Psoriasis). J Dermatolog Treat. (2024) 35:2393376. doi: 10.1080/09546634.2024.2393376, 39164008

[22] Phan C Beneton N Delaunay J Reguiai Z Boulard C Fougerousse AC . Effectiveness and safety of anti-interleukin-17 therapies in elderly patients with psoriasis. Acta Derm Venereol. (2020) 100:adv00316. doi: 10.2340/00015555-3678, 33111960 PMC9309849

[23] Talamonti M Russo F Malara G Hansel K Papini M Cattaneo A . Efficacy and safety of secukinumab in elderly patients with moderate to severe plaque-type psoriasis: *post-hoc* analysis of the SUPREME study. Clin Cosmet Investig Dermatol. (2023) 16:847–52. doi: 10.2147/CCID.S400520, 37033782 PMC10075320

[24] Megna M Camela E Battista T Genco L Martora F Noto M . Efficacy and safety of biologics and small molecules for psoriasis in pediatric and geriatric populations. Part II: focus on elderly patients. Expert Opin Drug Saf. (2023) 22:43–58. doi: 10.1080/14740338.2023.2173171, 36718748

[25] Hilhorst N Deprez E Pauwels N Grine L Lambert J Hoorens I. Patient-relevant outcomes in psoriasis: a systematic review. JAMA Dermatol. (2022) 158:806–11. doi: 10.1001/jamadermatol.2022.1756, 35675070

[26] Otuki MF Reis RC Cabrini D Prudente AS Horinouchi CD Correr CJ. Patient-reported outcomes in psoriasis research and practice. Br J Dermatol. (2011) 165:1361–2. doi: 10.1111/j.1365-2133.2011.10469.x, 21692769

[27] Yosipovitch G Tang MB. Practical management of psoriasis in the elderly: epidemiology, clinical aspects, quality of life, patient education and treatment options. Drugs Aging. (2002) 19:847–63. doi: 10.2165/00002512-200219110-00003, 12428994

[28] Ständer S Zeidler C Pereira M Szepietowski JC McLeod L Qin S . Worst itch numerical rating scale for prurigo nodularis: a psychometric evaluation. J Eur Acad Dermatol Venereol. (2022) 36:573–81. doi: 10.1111/jdv.17870, 34908192

